# Genetic risk scores to predict the prognosis of chronic heart failure patients in Chinese Han

**DOI:** 10.1111/jcmm.14722

**Published:** 2019-10-31

**Authors:** Shiyang Li, Yang Sun, Senlin Hu, Dong Hu, Chenze Li, Lei Xiao, Yanghui Chen, Huihui Li, Guanglin Cui, Dao Wen Wang

**Affiliations:** ^1^ Division of Cardiology Department of Internal Medicine Tongji Medical College Tongji Hospital Huazhong University of Science and Technology Wuhan China; ^2^ Hubei Key Laboratory of Genetics and Molecular Mechanisms of Cardiological Disorders Huazhong University of Science and Technology Wuhan China; ^3^ The First Affiliated Hospital of the Medical College Shihezi University Shihezi China

**Keywords:** chronic heart failure, exome sequencing, genetic risk score, inheritance

## Abstract

Chronic heart failure (CHF) has poor prognosis and polygenic heritability, and the genetic risk score (GRS) to predict CHF outcome has not yet been researched comprehensively. In this study, we sought to establish GRS to predict the outcomes of CHF. We re‐analysed the proteomics data of failing human heart and combined them to filter the data of high‐throughput sequencing in 1000 Chinese CHF cohort. Cox hazards models were used based on single nucleotide polymorphisms (SNPs) to estimate the association of GRS with the prognosis of CHF, and to analyse the difference between individual SNPs and tertiles of genetic risk. In the cohort study, GRS encompassing eight SNPs harboured in seven genes were significantly associated with the prognosis of CHF (*P* = 2.19 × 10^−10^ after adjustment). GRS was used in stratifying individuals into significantly different CHF risk, with those in the top tertiles of GRS distribution having HR of 3.68 (95% CI: 2.40‐5.65 *P* = 2.47 × 10^−10^) compared with those in the bottom. We developed GRS and demonstrated its association with first event of heart failure endpoint. GRS might be used to stratify individuals for CHF prognostic risk and to predict the outcomes of genomic screening as a complement to conventional risk and NT‐proBNP.

## 1 INTRODUCTION

Chronic Heart failure (CHF) is not simply the end‐stage of all types of heart disease but also a multifactorial epidemic disease. The mechanisms of CHF involve a complex interplay among neurohormonal,^1^ metabolic,^2^ genetic,^3^ inflammatory^4^ and other biochemical factors. Meanwhile, HF has a genetic predisposition that has been widely recognized by Framingham heart Study^5^ and Swedish Nationwide Adoption Study,^6^ in addition to the traditional risk factors. Genetic testing strategies might be a potential tool for individualized disease risk detection, prevention and intervention. Genetic risk scores (GRS), which is the sum of risk genes in individuals has been beneficial to primary prevention. Genome‐wide association study (GWAS) studies have identified 73 gene mutation loci for coronary artery disease (CAD),^7^ and almost 100 genes were associated with cardiomyopathies (most of which are DCM and HCM),^3^ while only few previous studies such as studies on coronary artery disease,^8^ and cardiometabolic diseases^9^ in cardiovascular disease have shown the use of genomic information in risk prediction.

Genetic variation in subjects with CHF may determine outcomes,^10^, ^11^ but previous studies on the prognosis of HF and related GRS remain unclear. First, CHF has broad spectrum of aetiology and heterogeneity of symptoms^12^; however, the common characteristics of genetics and/or environment have verified a final common pathway in CHF.^13^ Second, the outcomes of HF have cumulative effects for multiple risk factors interaction. In determining the prognosis of CHF, the role of monogenic variant is rare, and this will miss the superposition of minor genetic variations. Hence, there is a considerable room for improvement to genetic risk assessment for CHF.

Here, we reported a whole exome sequencing wide GRS for CHF to provide prognosis risk evaluation. We re‐analysed the mass spectrometry (MS) data for 34 non‐failing and failing human left ventricular myocardium.^14^ Moreover, in more than three phenotypes of failing heart, such genes will be included only if it has the same variability trend as normal contrast expression (*P* < 0.05), and thus were detected from whole exome sequencing data of 1000 Han Chinese CHF patients (787 idiopathic dilated cardiomyopathy and 213 ischaemic dilated cardiomyopathy). GRS for HF was constructed utilizing cox regression in HF cohort, to evaluate the stratifying prognosis and risk performance of GRS in 1000 CHF study cohort.

## 2 MATERIALS AND METHODS

### 2.1 Data of mass spectrometry of human myocardial tissue

Thirty four hearts samples were all diagnosed and collected by Chen from the Hospital of the University of Pennsylvania.^14^ The samples were divided into normal, compensated hypertrophy (cHyp), hypertrophic cardiomyopathy preserved ejection fraction (HCMpEF), hypertrophic cardiomyopathy reduced ejection fraction (HCMrEF), dilated cardiomyopathy (DCM) and ischaemic cardiomyopathy (ICM). The baseline of samples is available on https://www.nature.com/articles/ s41591‐018‐0046‐2#Sec33. Proteomic data of these patients can be publicly downloaded from proteomeXchange (http://www.proteomexchange.org/,PXD008934). The flow chart of the current study is provided in Figure S1.

### 2.2 Study subjects for whole exome sequencing

The Institutional Ethics Committee of Tongji Hospital approved this study, which followed the principles of the declaration of Helsinki. All subjects gave written informed consents before enrolment. At the cut‐off time in November 2017, 1000 patients (787 patients with dilated cardiomyopathy 15 and 213 patients with ischaemic dilated cardiomyopathy with left ventricular volume >60 mm and EF < 50%) from Cardiology Division of Tongji Hospital were enrolled. According the follow‐up protocol, all enrolled patients underwent face‐to‐face interviews or/and phone call interviews. Next, there was physical examination of clinic and ward patients by outpatient and attending physicians, respectively. The primary endpoint was defined as heart transplantation or cardiovascular death^10^, ^16^ that was confirmed using hospital death certificates or electronic medical records. Secondary endpoints were defined as heart failure readmission, discharge composites, and all‐cause mortality. Patients were followed up by specialized staff and the anthropometric measurements data, clinical characteristics and clinical events were electronically recorded via clinic visits and telephone calls. Baseline demographic and family history of all study participants were obtained via standardized questionnaires. All the laboratory examinations were executed using the Rocha modular DPP system according to standard procedures at the Department of Clinical Chemistry, Tongji Hospital. The rate of follow‐up compliance was 99.8% (998/1000), with 2% loss. The patients had a mean age of 57 years (57.0 ± 14.3), of which 25.7% were females. The clinical characteristics of individuals are summarized in Table 1. The risk factors were defined as follows: gender, age, hypertension, hyperlipemia, diabetes mellitus and current smoking. Meanwhile, β‐blocker taking as an adjusted factor was collected in different time points. Inclusion and exclusion criteria and the details of data processing and quality control are provided in Appendix S1.

**Table 1:** Baseline characteristics of whole exome sequencing population

Characteristics	Sequencing DCM population cohort (n = 1000)
Men	743 (74.30%)
Age, y	57.00 ± 14.19
NYHA	
II	296 (29.6%)
III	411 (41.10%)
IV	216 (21.60%)
LVEF (%)	34.55 ± 12.40
NT‐proBNP (pg/mL)	3750 (1555, 8645)
Glucose, mmol/L	6.80 ± 2.89
TC, mmol/L	3.91 ± 1.31
TG, mmol/L	1.40 ± 1.13
HDL, mmol/L	1.08 ± 3.33
LDL, mmol/L	2.42 ± 0.87
SBP, mm Hg	128.48 ± 40.62
DBP, mm Hg	80.65 ± 17.12
Hypertension	392 (39.20%)
Diabetes	175 (17.50%)
Hyperlipidemia	50 (5.00%)
Current smoking	390 (39.00%)
β‐blocker use	435 (43.50%)

Abbreviations: DBP, diastolic blood pressure; HDL‐C, high‐density lipoprotein cholesterol; LDL‐C, low‐density lipoprotein cholesterol; SBP, systolic blood pressure; TC, total cholesterol; TG, triglyceride.

### 2.3 Whole exome sequencing and bioinformatics workflow

Genomic DNA was extracted from peripheral blood leukocytes using Tiangen commercially available kit (Tiangen). Experimental workflow, sample preparation and sequencing were performed as protocol. All gDNA were of high quality and were determined through spectrophotometric and electrophoretic analyses. We fragmented genomic DNA to 300 bp sizes, and we used SureSelectXT exon V6 kit (Agilent) to capture target regions, repair fragments ends and ligate adapters. Following standard Illumina protocol, Illumina HiSeq X Ten sequencer was used to sequence the amplicons. Reads were aligned to hg19 human reference genome (GRCh37 Genome Browser) using Burrows‐Wheeler Alignment Tool (BWA) 0.7.17. The picard (http://picard.sourceforge.net) was used in removing duplicated reads. Insertions and deletions were recalibrated using GATK version 3.4. The variants exclusion criteria were low coverage (<20×), low quality score (<20) and low average quality (<3). Qualified samples were defined as variants of over 80% of the individuals that reached the read coverage of 20×. We used ANNOVAR to annotate the variants.

### 2.4 Data processing and quality control

The WES data were stored with Variant Call Format (VCF). The VCFtools (https://github.com/vcftools/vcftools) was used to perform data analysis, and invalid data were eliminated before establishing available data pools. Considering the repeatability of data processing, we employed appropriate quality control procedures to suit the whole exome sequencing summary statistics adapted for minor allele frequency. PLINK^16^ was used to control imputation quality and Hardy‐Weinberg equilibrium. Genetic principal components (PCs)^17^ was used for cohort structure quality control.

### 2.5 Construction of GRS

A detailed description is offered in Appendix S1. Briefly, (a) we built a GRS based on mass spectrometry of human myocardial tissue and exome sequence data from CHF subjects. (b) included loci of GRS were filtrated by Kaplan‐Meier analysis and the weighted sum of the risk allele(0/1/2) was supplied by Samuli Ripatti 18 and (c) logistic regression and C‐index (https://www.medcalc.org/ Medcalc‐version 18.11.3)^19^ were used for assessment of GRS.

### 2.6 Statistical analysis

To test the association of HF susceptibility variants with the prognosis of CHF risk factors**,** we used a combination of linear and logistic regression adjusting for age at first visit, gender, SNPs and conventional risk factors. These analyses were based on additive models. We considered the significant of SNP risk factor association threshold of *P* < 0.05. We analysed individual SNPs and tertiles of genetic risk score, which were adjusted using conditional logistic regression of GRS for age, gender and conventional risk factors, and the significance of the difference in the receiver operating characteristic (ROC) curves was tested with C‐index approach. Linkage disequilibrium (LD) was calculated by Haploview version 4.1. Data are expressed as mean ± SEM of experiments. Volcano plots (differential expression of genes for construction of GRS) were plotted by the R package ‘ggplot2.’ Data analyses were performed using SPSS 24.0 (SPSS, Inc) for Windows (Microsoft Corp).

## 3 RESULTS

### 3.1 Differential gene expression analysis

**Figure 1:**
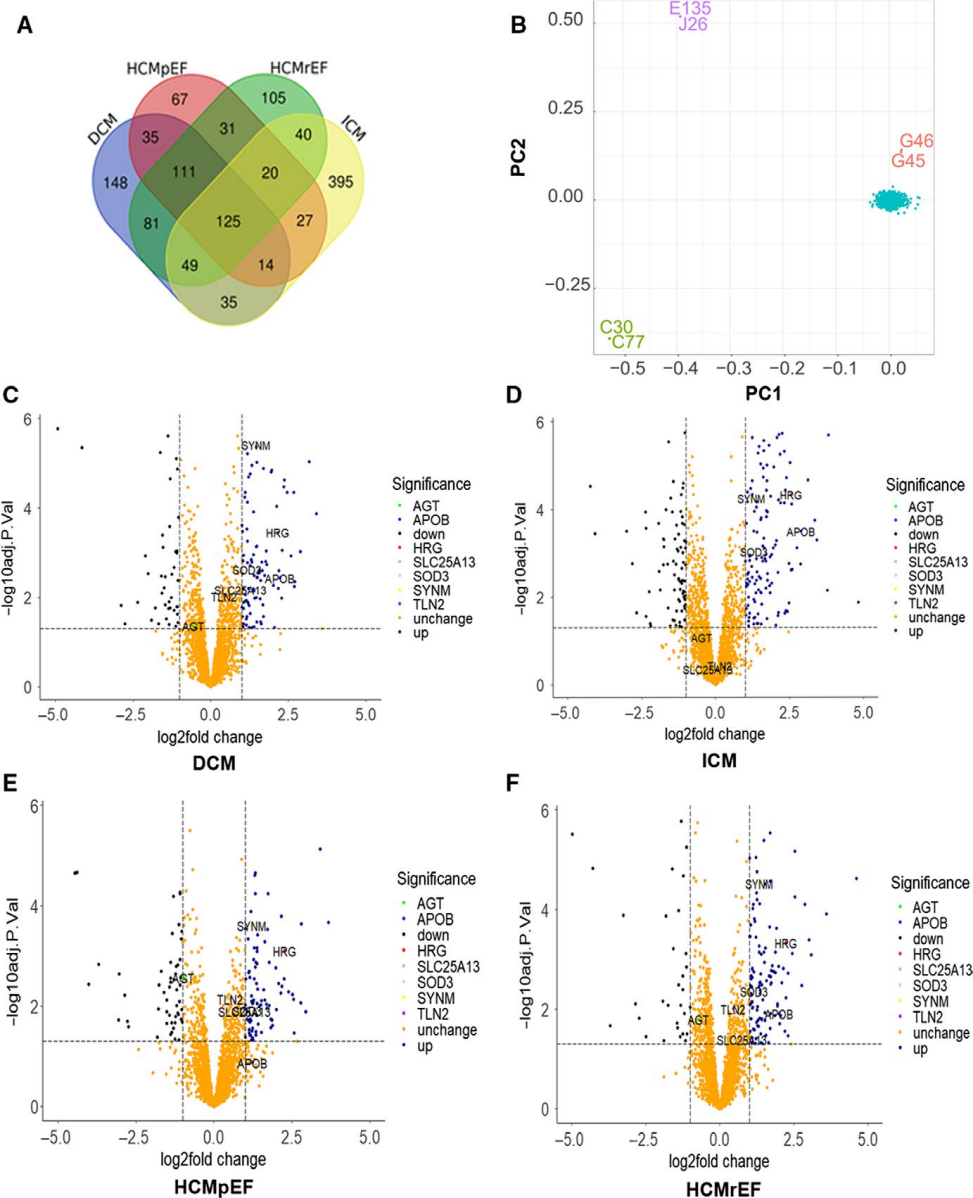
Combining mass spectrometry and whole exome sequencing to screen for target genes. A, Venn diagram to analyse the overlapping protein of using mass spectrometry of human myocardial tissue (Normal N = 7, ICM N = 6, HCMpEF N = 4, HCMrEF N = 5, DCM N = 6), 319 proteins were detected in more than three groups. B, Principal component analysis (PCA) of whole exome sequencing was performed to populations construction defined by Eigenstrata, the result demonstrated the consistency of the study (loadings of intermediates in PC1 and PC2 are shown in blue). C‐F, Volcano plot of genes used to build GRS in different phenotype of Mass spectrometry

In the online file (PXD008934), 3764 genes per group were available for analysis, and we filtered meaningful genes of *P* < .05 per group. The above conditions were for genes that were in more than three groups for HCMpEF, HCMrEF, DCM and ICM. Using Venn diagram to analyse the overlapping gene (Figure 1A), ultimately, 319 genes were chosen (details seen in Table S1). Baseline data for these samples are listed in https://www.nature.com/articles/s41591-018-0046-2#Sec33.

### 3.2 Whole exome sequencing

Seventy seven thousand, two hundred and eighty seven variants of Minor Allele Frequency (MAF) > 0.05 were identified in the whole exome sequencing from 1000 CHF objects, with 45 125 LD‐prune variants, which were used for the stratification of population (Figure 1B).

### 3.3 Differential gene loci and prognosis of patients with heart failure

To confirm the association between genetic alterations of common genes in the MS of heart failure tissues and the prognosis of CHF patients, 441 SNPs harboured in differentially expressed genes (Table S2) were identified by scanning the whole exome sequencing database consisting of 1000 CHF Chinese Han population. The primary outcomes occurred in 260 patients (26.0%). We selected only the lead SNP from each locus. In addition, we included SNP associated with the prognosis of HF, *P* < 0.05 if primary endpoints were the lead trait (most strongly associated) in Kaplan‐Meier analysis. Ten SNPs were identified among 441 variants in the additional model associated with the prognosis of HF (*P* < 0.05). After the identification of loci linkage (Figure S2), eight SNPs were verified to be associated with the prognosis HF (*P* < 0.05; Table 2, Table S7). The following genes *AGT*, *SLC25A13*, *HRG*, *APOB*, *SOD3*, *SYNM* and *TLN2* were included in the GRS study. In addition, volcano plot was used to display the variance of candidate genes among the different types of failing hearts (Figure 1C‐F).

**Table 2:** Association between SNPs and outcomes of heart failure

SNP	Gene	Chromosome	OMIM	Risk allele	MAF	*P* Value	HR (95% CI)
rs4273214	AGXT	2:240878862	604285	C	0.23	0.006	1.37 (1.09‐1.71)
rs33958047	AGXT	2:240878862	604285	G	0.18	0.005	1.36 (1.10‐1.68)
rs2301629	SLC25A13	7:96171508	603859	A	0.41	0.031	1.21 (1.02‐1.43)
rs1042464	HRG	3:186677783	142640	A	0.21	0.003	1.37 (1.11‐1.68)
rs679899	APOB	2:21028042	107730	G	0.15	0.045	1.26 (1.00‐1.59)
rs2536512	SOD3	4:24799693	185490	A	0.33	0.006	1.31 (1.08‐1.58)
rs3134587	SYNM	15:99 130 073	606087	T	0.30	0.009	1.28 (1.07‐1.54)
rs1320191	TLN2	15:62717605	603859	G	0.06	0.037	0.62 (0.39‐0.97)

Abbreviations: HR, hazard ratio; OMIM, Online Mendelian Inheritance in Man (http://www.omim.org/); SNP, single nucleotide polymorphism.

### 3.4 Predictive effect of GRS on the prognosis of HF

**Figure 2:**
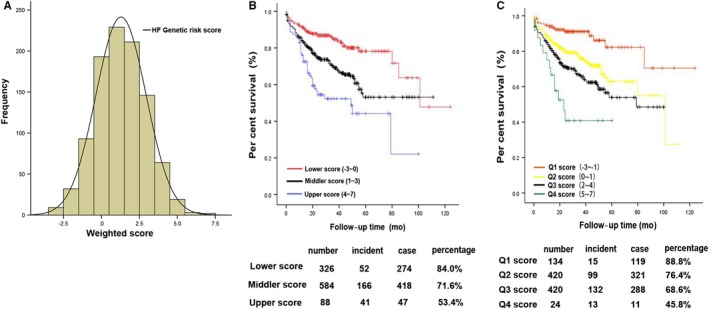
The distribution of GRS and combined effects of risk alleles on the prognosis of HF in prospective cohort study (A). For each subject, the number of risk alleles of eight replicated loci was summed to represent an individual's genetic risk score (range, −3 to 7). Individuals in each risk allele category are shown along the *X*‐axis, and *Y*‐axis on left represents the frequency of each genetic risk score category. (B, C) Cox proportional hazards model analysis after adjusted for gender, age, hypertension, hyperlipemia, diabetes mellitus, current smoking and β‐blocker treatment, showed the association of GRS with cardiovascular deaths or cardiac transplantation in the tertiles of genetic risk score (B, HR = 3.68, 95% CI 2.40‐5.65, *P* = 2.47 × 10^−10^) and quartiles (C, HR = 6.76, 95% CI 3.21‐14.28, *P* = 5.27 × 10^−7^)

For each individual, we calculated CHF‐specific genetic scores using the weighted sum of the risk allele (zero, one, or two for risk alleles at each locus; Figure 2A). These scores were weighted according to the size effect reported in the endpoints studies. Genetic risk score was strongly associated with the prognosis of CHF by univariable analysis (HR = 1.28, 95% CI 1.19‐1.37, *P* = 5.16 × 10^−11^) and multivariable analysis including adjusted traditional risk factors and β blocker taking (HR = 1.28, 95% CI 1.18‐1.37, *P* = 2.19 × 10^−10^) by Cox Proportional Hazard Analyses (Table 3).

**Table 3:** Results of univariable and multivariable cox proportional hazard analyses for cardiac events

Variables	Univariable analysis^a^			Multivariable analysis^b^		
	HR	95% CI	*P* value	HR	95% CI	*P* value
Gender	1.35	1.04‐1.76	0.03	1.23	0.93‐1.62	0.15
Age	1.03	1.02‐1.04	1.18 × 10^−8^	1.02	1.01‐1.03	4.54 × 10^−4^
Hypertension	1.10	0.86‐1.41	0.46	1.19	0.91‐1.55	0.20
Diabetes	0.77	0.57‐1.03	0.07	0.76	0.56‐1.04	0.09
Hyperlipidemia	0.95	0.74‐1.21	0.66	0.95	0.75‐1.19	0.63
Current smoking	1.09	1.01‐1.17	0.02	1.09	1.00‐1.18	0.05
β‐blocker use	5.82	4.03‐8.40	5.43 × 10^−21^	5.48	3.78‐7.93	2.07 × 10^−19^
Genetic risk score	1.28	1.19‐1.37	5.16 × 10^−11^	1.28	1.18‐1.37	2.19 × 10^−10^

a HR, Hazard ratios and* P* value were calculated with univariate cox proportional hazard model.

b HR, Hazard ratios and* P* value were calculated with the use of cox proportional hazard model adjusted gender, age and traditional risk factor: hypertension, hyperlipemia, diabetes mellitus, current smoking.

The tertiles of GRS were utilized in establishing model 1 (original risk), model 2 (age and gender) and model 3 (age, gender and traditional risk factors^20^) with details provided in Table S3. The consistency of estimates for the models did not change when adjustment for traditional risk factors was compared with the original risk only. Participants in the top tertiles of genetic risk score were estimated to have 3.56‐times increased risk of primary outcomes compared with those in the bottom tertiles (95% CI 2.36‐5.37, *P* = 1.34 × 10^−9^). After adjustment for age, gender and traditional risk factors, the HRs remained the same, with 3.68‐times expansion (95% CI 2.40‐5.65, *P* = 2.47 × 10^−10^; Figure 2B, Table S4).

When the genetic risk score was divided into quartiles as previous tertiles, results were consistent and associated with the prognosis of CHF; details are provided in Table S3. The top quartile of the genetic risk score has 6.96 times increased risk compared with those in the bottom quartiles of the original risk (95% CI 3.30‐14.68, *P* = 3.44 × 10^−7^). After we adjusted the traditional risk factors and the β‐blocker taking, HR acquired 6.76 times expansion (95% CI 3.21‐14.28, *P* = 5.27 × 10^−7^; Figure 2C, Table S4).

**Figure 3:**
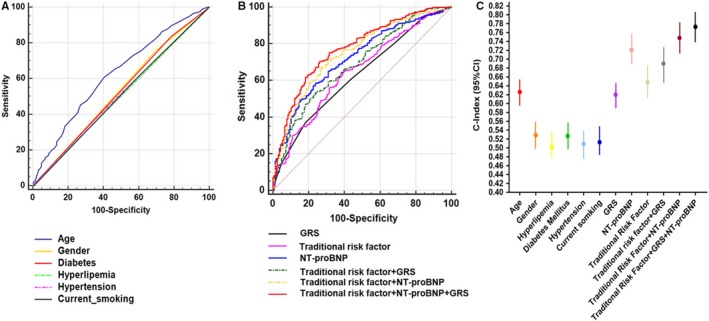
Predictive outcomes of HF using the GRS and conventional risk factors. A and B, Receiver operating characteristic (ROC) analyses were performed to individual of traditional risk factors, and compound factors. C, C‐index for Cox regression of incident HF with individually traditional risk and in combination factors was illuminated that the model, traditional risk combining NT‐proBNP and GRS, has better predictive ability compared with others

To compare GRS predictive validity to traditional risk factor, we constructed a risk prediction model that included gender, age, hypertension, hyperlipemia, diabetes mellitus and current smoking. By employing binary logistical regression to analyse the traditional risk factors, a predictive indicator was taken for the receiver operating characteristic (ROC) curves. The N‐terminal B‐type natriuretic peptide (NT‐proBNP) was built as a contrast scale, which has been widely recognized as an important prognostic indicator of heart failure.^21^, ^22^, ^23^ NT‐proBNP in GRS of tertiles and quartiles showed no differentiation with ANVOA (*P* = 0.895; *P* = 0.704; Table S8). In Cox regression of prognosis of CHF, models based on age had higher C‐index (*C* = 0.626; 95% CI: 0.595‐0.656) than any of the individual conventional risk factors, with the second‐best model being GRS on assessment (*C* = 0.620; 95% CI: 0.589‐0.650; Figure 3A and 3). A model combining these seven traditional risk factors (TRA), (*C* = 0.648; 95% CI: 0.610‐0.684) had only slightly better performance than GRS (*P* = 0.592; 95% CI: −0.0486‐0.852). Combining the GRS with TRA resulted in a model with C‐index of 0.690 (95% CI: 0.653‐0.725), an increase of 4.9% over the model consisting of only TRA, and a significant difference (*P* = 0.017; 95% CI: −0.00759‐0.0773). A model combining TRA, GRS and NT‐proBNP (*C* = 0.773; 95% CI: 0.740‐0.804) has better predictive ability, and an increase of 7.2% (*P* = 0.0016; 95% CI: 0.0198‐0.0847), compared with the use of only NT‐proBNP model (*C* = 0.721; 95% CI: 0.685‐0.755). The result showed an amplification of 3.34%, with significant difference (*P* < 0.0001; 95% CI: 0.0488‐0.118) for the combination of TRA, NT‐proBNP and GRS models compared with combined models of TRA and NT‐proBNP (*C* = 0.748; 95% CI: 0.713‐0.781; Figure 3B and C, Tables S5 and S6).

## 4 DISCUSSION

Genetic counselling is recommended for CHF patients and their family members.^24^ It has always been of clinical focus to foresee the risk of heart failure via genetic background characteristics. GRS was used to identify patients with Mendelian and complex disease^25^ patterns known as loci for HF risk factors, such as CAD, Cardiometabolic Disease,^8^, ^26^ Blood Pressure^9^ and atrial fibrillation.^27^ Heart failure has similar or worse prognosis when compared to most cancers.^28^ Traditional risk factors^20^ have been used in risk prediction. However, obtaining GRS to predict the prognosis of HF remains challenging. Here, we made an effort to incorporate genetic risk scores into clinical practice for determining the outcome of CHF.

In analysing the data from case‐control mass spectrometry of human left ventricle tissue in this cohort study, 441 genes were used to search for common variant loci (*P* > 0.05), and whole exome sequencing was performed for 1000 Chinese Han population with HF. We aimed to validate differentially expressed genes to estimate the association of outcomes with the magnitude of risk conferred by these genetic risk factors in the population setting. Seven genes were used for GRS construction: Alanine–glyoxylate and serine–pyruvate aminotransferase (AGXT), Solute carrier family 25 member 13 (SLC25A13), Histidine‐rich glycoprotein (HRG), Apolipoprotein B (ApoB), SOD3, Synemin (SYNM) and TLN2. AGXT are mostly localized in the peroxisomes, and may be associated with primary hyperoxaluria type 1.^29^ SLC25A13 encodes aspartate/glutamate carrier isoform 2 (AGC2) and involves in numerous metabolic pathways including energy metabolism pathway and cell functions.^30^ HRG has two cystatin‐like domains, located in 3q27 on the chromosome 3 by binding different ligands to modulate various biological processes including angiogenesis, coagulation and immune function.^31^ ApoB refers to heart and vascular diseases, having a product of primary apolipoprotein of chylomicrons and VLDL, and it is the primary organizing protein component of the particles.^32^ SOD3 encoding a member of superoxide dismutase (SOD) protein family is an antioxidant enzyme that catalyses the conversion of superoxide radicals into hydrogen peroxide and oxygen, which may protect the heart from oxidative stress.^33^ SYNM is an intermediate filament (IF) family member, which primarily functions as mechanical stress, maintains structural, and related to smooth muscle cell cytoskeleton of the heart, with its absence causing ventricular dysfunction in mice.^34^ TLN2 belonging to the talin protein family is a cytoskeletal protein, which is highly expressed in cardiac muscle when loss of talin‐1 and talin‐2 leads to dilated cardiomyopathy and cardiac dysfunction in mice.^35^ Clinicians have a growing need for tools to assess the prognosis of heart failure accurately in the individual patient, especially to obtain credible information regarding the prognosis in the early stage and not just collecting clinical characteristics of symptoms and the results of invasive test in the acute exacerbation stage. SNPs as factors of the innate genetic background are involved in the pathophysiological processes of heart failure. This is a new attempt to build a genetics risk score consisting of SNPs to predict the outcomes of heart failure. We found that genetic risk score including eight SNPs (rs4273214, rs33958047, rs2301629, rs1042464, rs679899, rs2536512, rs3134587 and rs1320191) harboured seven genes were associated with heart failure (even after we have accounted traditional risk factors and β blocker taking). In addition, the top 1/3 of individuals of Chinese Han ancestry who had the most risk alleles have roughly 3.87 times risk to develop the incidence of cardiac death and heart transplantation compared with those in the lowest tertiles. The GRS of HF had a very good predicting ability to categorize individual patients in separate risk strata.

Several ways have been widely accepted to estimate the prognosis of heart failure. The Seattle prediction model used common clinical characteristics to predict accurately the survival of heart failure patients with large samples. ROC curve of the model was 0.729 (95% CI, 0.714‐0.744), but it was limited by multivariable and without NT‐proBNP.^36^ CHARM‐model including 21 predictor variants has a similar performance with C‐index of 0.74 and 0.76 for cardiovascular death in low and the preserved left ventricular EF populations.^37^ Its highlights showed non‐invasive indicators and accuracy of stratify risk to classify participators. However, previously mentioned model did not utilize orthodox biochemical markers such as NT‐proBNP or BNP to construct risk model. The MUSIC risk score is a high accuracy model with preserved LVEF cases,^38^ including NT‐proBNP and other 10 risk factors which have strong discrimination to predict total mortality; cardiac mortality and C‐indices were 0.77 and 0.78. The advantages of MUSIC model are simple operation and identification of high‐risk patients; however, it has reduced LVEF patients and traditional predictors, which could impede the application to general patients.

Because of the poor outcomes and changing risks in different phases of HF, clinicians might draw differential predictions from the information of patient characteristics that were not readily available and alterable with the stage and severity of HF. Meanwhile, the risk factors used to build classical prediction models, usually included over 10 risk factors of clinical test and clinical characters, which have restriction in assessing doctors and hospitals. However, our clinical predictive model only needs one gene panel, numerous of traditional cardiovascular risk factors to make predictions and has better performance when combined with NT‐proBNP. We evaluated the performance of GRS validation in the Chinese Han heart failure population. Genetics risk score was strongly associated with the prognosis of CHF by univariable and multivariable in Cox Proportional Hazard Analyses (HR = 1.28, 1.28; *P* = 5.16 × 10^−11^, *P* = 2.19 × 10^−10^). The C‐index was 0.620. GRS model combined with NT‐proBNP and traditional risk factors was highly discriminatory and improved specificity and accuracy in identifying HF patients with worse prognosis (C‐index is 0.773). The predictive power of GRS + TRS + NT‐proBNP model is not inferior to the classical models.

The following conclusions can be drawn from our findings. First, we reported a series of findings that attempted to stratify individuals with HF using genomic information prognosis in general populations, an approach that leverages the fixed nature of germline DNA over the life course to anticipate different lifelong trajectories of CHF. Second, the variants from case‐control differentially expressed genes appeared to affect the outcomes, including those from whole exome sequencing prospective cohorts. Third, further adjustment for traditional risk factors had no effect on the risk estimates because of the genetic risk score. Finally, genetic risk score combined with traditional risk factors improved risk discrimination when assessed with the C‐index (*P* = 0.017), and this is strongly associated with the outcomes of HF.

Overall, this is a challenge to any single risk factor to predict prognosis of CHF via complex traits. Our combined results showed much powerful risk discriminations between the tails of genetic risk scores. The differences cannot be accounted by NT‐proBNP as they are similar for each score in our study. Hence, higher genetic risk indicating individuals that have more genetic risk alleles has worse prognosis even if they have the same level of NT‐proBNP. Subdividing patients can help us identify high‐risk individuals more accurately. For clinical practice, providing precise interventions for variants subjects in early stage of CHF (stage A or B) would improve benefits. This potential value of using genetic risk score and optimizing the use of scarce resources for the estimation of the endpoint of CHF would be revalidated in further studies.

### 4.1 Study limitations

First, the SNPs used for GRS panel construction should be enriched. For example, more potential differential genes can be obtained after augmenting the sample size of MS control‐case. Meanwhile, extensive whole exome sequencing cohort potential candidate variants will unfold with GRS. Second, there are racial differences between mass spectrometry (MS) samples and whole exome sequencing population. Another population or other ancestries are needed to verify the results. However, the potential clinical use of GRS can be predicted. Third, the expansion of the sample size of CHF population is needed, and future studies on large multiethnic cohorts will validate GRS. Fourth, other widely accepted heart failure prognostic risk scores, such as Seattle heart failure model should be included as control in subsequent studies. Fifth, we did not include the index of family history in traditional risk. The information about disease and its direct relations is often vague, which may be related to the level of medical care in the past.

## 5 CONCLUSION

We developed and assessed a genetic score based on eight SNPs in the current study, and we demonstrated that it was associated with the first event of heart failure endpoint. We attempted the concept of engaging genomic information to stratify individuals for CHF prognostic risk and to improve risk reclassification for participants, and we demonstrated a hypothesis to predict the outcomes of genomic screening to complement conventional risk and NT‐proBNP.

## CONFLICT OF INTEREST

The authors declare no competing financial interests.

## AUTHOR CONTRIBUTIONS

Shiyang Li and Senlin Hu developed the study concept, design and interpreted the data and drafted the manuscript. Dong Hu and Sun Yang performed the data analysis. Lei Xiao, Yanghui Chen, Huihui Li performed the research, Guanglin Cui, Dao Wen Wang supervised the design of the study and revised the manuscript.

## Data Availability

The authors confirm that the data supporting the findings of this study are available within the article and its supplementary materials.
