# Supplementary material for: Genetic risk scores to predict the prognosis of chronic heart failure patients in Chinese Han
Source: J Cell Mol Med. 2019 Oct 31;24(1):285–93. doi: 10.1111/jcmm.14722 (PMC6933418; doi:10.1111/jcmm.14722)
Supplement: Supplementary file 1 [file JCMM-24-285-s001.doc]

Supplementary Materials for

**Genetic Risk Prediction of Prognosis in Chinese Han Chronic Heart Failure Patients**

Shi-Yang Li, MD, Yang Sun, MD,Sen-Lin Hu, MD, Dong Hu MD, Chen-Ze Li, MD, Lei-Xiao, MD, Yang-Hui Chen, MD, Hui-Hui Li, MD, Jing-Wang, MD, Guang-Lin Cui, MD, PhD and Dao-Wen Wang, MD, PhD

**1.Recruitment for the heart failure cohort**

We used data from one independent study pool comprising up to 1000 participants of Chinese Han. The diagnosis of HF according to the guideline of ACC/AHA ^1^, and DCM according to WHO/ISF^2^. Ischemic cardiomyopathy defined as mild coronary heart disease (left ventricular volume >60 mm and ejection fractions <50%). Hypertension was systolic >140 mmHg, and or diastolic >90 mmHg or current treated with an antihypertensive drug. Diabetes was defined fasting blood-glucose > 7.8 mmol/L, plasmaglucose >11.1 mmol/L, Hyperlipidemia was assessed as total plasma cholesterol >5.72 mmol/L or plasma triglyceride >1.70 mmol/L. Collecting Vein blood specimen after an overnight fast were stored frozen at -80°C. Laboratory examination was executed from DPP system according to standard procedures at the Department of Clinical Chemistry, Tongji Hospital. This study was approved by the institutional ethics committees of the Tongji hospitals, and written informed consent was obtained from all study participants. Diagnosis were done by professional cardiologist via history-taking, physical examination, laboratory test, and echocardiography. We only included subjects who independently accomplished standardized questionnaire and have electronic medical record. The exclusion criteria from the study met under the following conditions: resistant hypertension, severe valvar disease and ischemic heart disease, fatal systemic infection, excessive alcohol consumption, thyroid-induced cardiomyopathy, previous malignancy history, or patients refused to participate in the follow-up. The status of clinical risk factors which such as family history, smoking, past disease history is collected by structured questionnaires

**2.Construction of the GRS for heart failure**

To construct a genomic risk score (GRS) for the prognosis HF, we followed a bioinformatic analysis strategy using the mass spectrometry (MS) data from 34 non- failing and failing human left ventricular myocardium, which focused on the functional effects of microtubule detyrosination in heart failure. The MS data not only to reveal the up-down regulation of intermediate filaments and microtubules but unfolded a map for variational of proteomic^3^. Proteomic data of these patients can be publicly downloaded at proteomexchange ([http://www.proteomex](http://www.proteomex/)change.org/,PXD008934).

Firstly, we established a pool of proteomics variation which is composed with Genes that in more than three types of heart failure (HCMpEF, HCMrEF, DCM, ICM) have differential expression(p<0.05). Next, we performed exome sequencing for the cohort included 1000 Han Chinese HF patients treated at TongJi Hospital. We obtained the variant spectrum of the above genes. SNPs associated with outcomes of HF tested with the Cox proportional hazards model adjusted for gender, age, hypertension, hyperlipemia, diabetes mellitus, current smoking, β-blocker treatment. Meanwhile, we identified linkage allele using Haploview. Thirdly, we constructed a multilocus genetic risk score for each individual by summing the number of risk alleles (0/1/2) for each of the 8 SNPs weighted by their estimated effect sizes in the discovery sample.


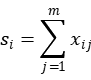

where
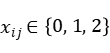
 is the count of the minor allele for the jth variant in the ith individual.

| **S1 Table enrolled 319 genes by veen analysis in mass spectrometry of human myocardial tissue** | | |
| --- | --- | --- |
| **Names** | **total** | **elements** |
| DCM HCMpEF HCMrEF ICM | 125 | ACTN4 MAP4 SLC4A1 HSPB6 UQCR11 C8A HRG SERPINA6 DPYSL2 GOT1 PRDX6 AMBP PALLD DKFZp686G11190 UCHL1 XIRP1 LGALS1 APOE SOD3 DDAH1 COL18A1 LBP IDH2 TF SND1 SPTAN1 CLEC3B CRP HBG2 IGLV3 FKBP4 PGLYRP2 CD151 SCAMP3 CTSB SVIL RPL7 TUBA1A IGK@ VIM WARS DES HSPA2 HBD IGLL5 NDUFAF7 FKBP5 VKA17 CRAT PFKM CDS2 PPP1R12C S100A6 RPL6 AZU1 SERPIND1 GPT PDK4 SERPINA4 HSP90AA1 EEF1A2 SYNPO2L CD163 PGP LUM EEF2 MFAP4 CSDC2 TTR NAMPT ACY1 RBP4 MFGE8 IGKV1 V3 SUMO2 PDLIM1 EIF5 THBS4 ARHGAP1 CLU DKFZp686M08189 EHD3 PPIF COL14A1 A1BG AEBP1 ITIH2 CA2 NID2 SCRN3 SAA1 HBB KNG1 IGHV3OR16-9 CA3 CMBL PLG VCL PTGFRN HSPA1B CACYBP TMEM120A PDLIM3 NES AHSG PGM5 MINOS1 APOA4 SIAE SGCE F12 HIBADH ACTN1 PYGM PGAM2 CA1 SYNM TGFBI GATM CKM IGKV1-5 APOC1 HBA2 HEL |
| DCM HCMpEF HCMrEF | 111 | SLMAP GCSH DECR1 RPL7A ECH1 FAHD1 SLC44A2 PDCD5 PLN ALDH3A2 TPI1 DSTN SRI SLC25A5 QDPR MGST3 CDIPT YWHAQ CRYAB MYH6 RTN3 ELF3 GPI SPTBN1 SAMM50 RPS27A AGT RHOA HSPA8 HP1BP3 TSN HDHD2 RPL4 FABP3 SLC25A13 JPH2 CNDP2 SNRPD1 ATP5H RPL22 CTBP1 AGK PCYOX1 PRPS1 ACO1 RRAS PRDX5 SEC22B UBE2L3 ALDOA ANXA5 PSME1 H1F0 TP53I3 YWHAB SPEG PRDX3 HNRNPD MARCKS EIF1B TLN2 INPP4B GHITM HMGN2 NME2 UGDH MAPK1 MB HSPA9 RPS15A CYB5R1 RPL15 RPL3L RPS3A LGALS3 MXRA7 DBI SLC25A11 RAB4A AKR1B1 NIT1 CYB5R3 ARF1 ARF3 PTGES2 ENO3 RPL23 PSMD1 PURB CHCHD6 TOMM40L DDTL PKM CANX LRG1 DMD CISD1 TPPP DCTN2 PSME2 OLA1 SERPINA3 GSTM2 MIEN1 LACTB CUTA MTFR1L PEBP1 PEPD 8-Sep CKAP4 PGK1 |
| DCM HCMpEF ICM | 14 | GJA1 IMPDH2 ACTC1 HSPB2 TJP1 SH3GL1 CHST9 IGHG1 METAP2 SMTN TPM2 RAB1A RPL23A IGKV4-1 |
| DCM HCMrEF ICM | 49 | GALK1 SUSD2 SERPINF1 IGKV3-20 TGM2 ITGB1BP2 C3 FABP5 PARVA HSD17B4 MRPL28 APOB CFH TECR PCCB FHL1 ATP1A1 RRAS2 LTBP2 TPT1 COQ10A STAT3 DDAH2 CTSD ADSSL1 APEX1 BCL2L13 ALDH5A1 COX6A1 ACSS3 TUBA4A LAMB1 SORBS1 FSCN1 ABAT ACSL1 IGHA1 PSMB4 C21orf33 HSPH1 ITIH1 VCAN TUBB4B ANKRD1 APOA1 CKMT2 GAPDH SEC61B COMT |
| HCMpEF HCMrEF ICM | 20 | ANXA3 RPS23 MDH1 APEH NT5C STBD1 AAMDC FLOT2 DDX39A MPI HEXB PTER PDXP SRL PPP1R13L TUBB6 STIP1 DCPS APOA1BP DPYSL3 |
| &. Proteomic data downloaded from proteomexchange (http://www.proteomexchange.org/,PXD008934).  # Statistical significance was determined p<0.05 for each group, candidated genes for Veen analysis (http://bioinformatics.psb.ugent.be/webtools/Venn/) *Gene was selected in more than 3 groups. | | |

| **S2 Table common SNPs data queried from Whole Exome Sequencing** | | | | | | |
| --- | --- | --- | --- | --- | --- | --- |
| Chromosome | Position | GENE | ID | REF | Risk Allele | **MAF** |
| chr1 | 161196166 | TOMM40L | rs3813628 | A | C | 0.49 |
| chr1 | 117529458 | PTGFRN | rs10801922 | G | A | 0.34 |
| chr1 | 117503940 | PTGFRN | rs34625170 | G | A | 0.40 |
| chr1 | 156640156 | NES | rs3748570 | G | A | 0.12 |
| chr1 | 156641537 | NES | rs951781 | C | T | 0.11 |
| chr1 | 156639636 | NES | rs11549292 | G | A | 0.27 |
| chr1 | 156639753 | NES | rs11582300 | G | A | 0.13 |
| chr1 | 156640308 | NES | rs3748571 | C | T | 0.27 |
| chr1 | 156640503 | NES | rs3828043 | C | T | 0.27 |
| chr1 | 156640678 | NES | rs2886443 | G | A | 0.13 |
| chr1 | 85816134 | DDAH1 | rs2230820 | T | C | 0.16 |
| chr1 | 19950062 | MINOS1-NBL1 | rs1737428 | T | C | 0.19 |
| chr1 | 57340727 | C8A | rs652785 | C | A | 0.45 |
| chr1 | 57378149 | C8A | rs1620075 | G | T | 0.06 |
| chr1 | 57378150 | C8A | rs1620073 | C | T | 0.06 |
| chr1 | 57378198 | C8A | rs1754533 | T | C | 0.06 |
| chr1 | 57383315 | C8A | rs1342440 | G | C | 0.06 |
| chr1 | 57383358 | C8A | rs17300936 | C | T | 0.07 |
| chr1 | 151372047 | PSMB4 | rs2296840 | C | T | 0.39 |
| chr1 | 151372138 | PSMB4 | rs7172 | G | A | 0.22 |
| chr1 | 151374025 | PSMB4 | rs4603 | T | C | 0.39 |
| chr1 | 196642233 | CFH | rs800292 | G | A | 0.38 |
| chr1 | 196654324 | CFH | rs1061147 | A | C | 0.06 |
| chr1 | 196659237 | CFH | rs1061170 | C | T | 0.05 |
| chr1 | 196682947 | CFH | rs2274700 | G | A | 0.40 |
| chr1 | 196695742 | CFH | rs3753396 | A | G | 0.48 |
| chr1 | 196709774 | CFH | rs1065489 | G | T | 0.48 |
| chr1 | 201981218 | ELF3-AS1 | rs11543979 | C | G | 0.37 |
| chr1 | 201981862 | ELF3 | rs2819362 | T | C | 0.22 |
| chr1 | 202935911 | CYB5R1 | rs2232842 | T | C | 0.42 |
| chr2 | 54844790 | SPTBN1 | rs2229506 | C | T | 0.05 |
| chr2 | 54858511 | SPTBN1 | rs1052788 | C | T | 0.27 |
| chr2 | 54858664 | SPTBN1 | rs2229503 | C | T | 0.09 |
| chr2 | 54886347 | SPTBN1 | rs1052820 | G | A | 0.30 |
| chr2 | 220285309 | DES | rs1058261 | C | T | 0.18 |
| chr2 | 220286142 | DES | rs1058284 | G | A | 0.18 |
| chr2 | 21225281 | APOB | rs1042034 | C | T | 0.27 |
| chr2 | 21231387 | APOB | rs2163204 | T | G | 0.07 |
| chr2 | 21231524 | APOB | rs676210 | G | A | 0.27 |
| chr2 | 21232195 | APOB | rs693 | G | A | 0.06 |
| chr2 | 21232804 | APOB | rs1041968 | G | A | 0.06 |
| chr2 | 21250914 | APOB | rs679899 | G | A | 0.15 |
| chr2 | 21252534 | APOB | rs13306194 | G | A | 0.12 |
| chr2 | 21260084 | APOB | rs13306198 | G | A | 0.06 |
| chr2 | 21263900 | APOB | rs1367117 | G | A | 0.12 |
| chr2 | 65315711 | RAB1A | rs2227268 | G | A | 0.25 |
| chr2 | 70485320 | PCYOX1 | rs11556184 | C | T | 0.18 |
| chr2 | 220315970 | SPEG | rs10932806 | G | A | 0.31 |
| chr2 | 220330804 | SPEG | rs1567487 | T | C | 0.31 |
| chr2 | 220337041 | SPEG | rs116911250 | G | A | 0.09 |
| chr2 | 220348751 | SPEG | rs10755037 | C | T | 0.13 |
| chr2 | 220353013 | SPEG | rs875098 | G | A | 0.13 |
| chr2 | 220353532 | SPEG | rs13026308 | C | A | 0.06 |
| chr2 | 220354108 | SPEG | rs55760516 | A | G | 0.24 |
| chr2 | 220354365 | SPEG | rs56132883 | C | T | 0.13 |
| chr2 | 220354542 | SPEG | rs55969678 | G | A | 0.12 |
| chr2 | 220355529 | SPEG | rs12464085 | A | G | 0.16 |
| chr2 | 220356520 | SPEG | rs12473286 | G | T | 0.12 |
| chr2 | 220358198 | SPEG | rs1050816 | C | T | 0.24 |
| chr2 | 241808347 | AGT | rs34885252 | A | G | 0.08 |
| chr2 | 241812428 | AGT | rs117195882 | C | A | 0.08 |
| chr2 | 241812461 | AGT | rs34664134 | G | A | 0.08 |
| chr2 | 241813453 | AGT | rs33958047 | G | A | 0.18 |
| chr2 | 241817516 | AGT | rs4426527 | A | G | 0.08 |
| chr2 | 241818279 | AGT | rs4273214 | C | A | 0.23 |
| ch3 | 133465201 | TF | rs8177186 | G | T | 0.26 |
| ch3 | 133465283 | TF | rs1130459 | A | G | 0.23 |
| ch3 | 133474328 | TF | rs12769 | G | A | 0.45 |
| ch3 | 133475722 | TF | rs1799852 | C | T | 0.24 |
| ch3 | 133486958 | TF | rs8649 | G | C | 0.06 |
| ch3 | 133494354 | TF | rs1049296 | C | T | 0.27 |
| ch3 | 57882601 | SLMAP | rs17058639 | C | T | 0.48 |
| ch3 | 47956424 | MAP4 | rs1137524 | C | T | 0.29 |
| ch3 | 47958037 | MAP4 | rs1060407 | G | T | 0.26 |
| ch3 | 186435370 | KNG1 | rs1050274 | G | A | 0.30 |
| ch3 | 186437944 | KNG1 | rs5029980 | T | C | 0.06 |
| ch3 | 186440243 | KNG1 | rs1469859 | G | A | 0.24 |
| ch3 | 186443018 | KNG1 | rs1656922 | T | C | 0.37 |
| ch3 | 186445052 | KNG1 | rs2304456 | T | G | 0.17 |
| ch3 | 186459927 | KNG1 | rs710446 | T | C | 0.31 |
| ch3 | 186389559 | HRG | rs10770 | T | C | 0.10 |
| ch3 | 186390627 | HRG | rs9898 | C | T | 0.46 |
| ch3 | 186395113 | HRG | rs2228243 | A | G | 0.40 |
| ch3 | 186395436 | HRG | rs1042445 | C | T | 0.31 |
| ch3 | 186395572 | HRG | rs1042464 | A | T | 0.21 |
| ch3 | 45077123 | CLEC3B | rs13963 | G | A | 0.11 |
| ch3 | 186330969 | AHSG | rs4831 | C | G | 0.11 |
| ch3 | 186337713 | AHSG | rs4917 | T | C | 0.27 |
| ch3 | 186338425 | AHSG | rs1071592 | A | C | 0.18 |
| ch3 | 23963183 | RPL15 | rs9755750 | G | A | 0.10 |
| ch3 | 49714225 | APEH | rs1131095 | T | C | 0.06 |
| ch3 | 52819119 | ITIH1 | rs117815982 | G | A | 0.08 |
| ch3 | 52820981 | ITIH1 | rs678 | A | T | 0.36 |
| ch3 | 52821011 | ITIH1 | rs1042779 | A | G | 0.38 |
| ch3 | 52825528 | ITIH1 | rs1075653 | T | C | 0.37 |
| ch3 | 52825585 | ITIH1 | rs9324 | T | C | 0.37 |
| ch3 | 126451937 | CHCHD6 | rs2272487 | G | T | 0.32 |
| chr4 | 41258970 | UCHL1-AS1 | rs11556271 | A | G | 0.17 |
| chr4 | 41258978 | UCHL1-AS1 | rs9321 | C | T | 0.17 |
| chr4 | 41259633 | UCHL1-AS1 | rs5030732 | C | A | 0.46 |
| chr4 | 24801315 | SOD3 | rs2536512 | G | A | 0.33 |
| chr4 | 24801354 | SOD3 | rs8192291 | C | T | 0.39 |
| chr4 | 169432841 | PALLD | rs1806729 | G | A | 0.18 |
| chr4 | 169433326 | PALLD | rs7655494 | T | C | 0.18 |
| chr4 | 169433327 | PALLD | rs7671781 | G | A | 0.18 |
| chr4 | 169433333 | PALLD | rs7673220 | C | T | 0.18 |
| chr4 | 169606649 | PALLD | rs62333891 | C | A | 0.08 |
| chr4 | 169630204 | PALLD | rs58395080 | T | G | 0.33 |
| chr4 | 169799448 | PALLD | rs62333013 | A | G | 0.44 |
| chr4 | 169835134 | PALLD | rs1059444 | A | G | 0.05 |
| chr4 | 169432673 | PALLD | rs61051061 | C | T | 0.18 |
| chr4 | 122617745 | ANXA5 | rs1131239 | G | A | 0.17 |
| chr4 | 17503433 | QDPR | rs3733570 | C | T | 0.45 |
| chr4 | 17506042 | QDPR | rs12645938 | G | A | 0.06 |
| chr4 | 39501722 | UGDH | rs200660972 | CAAAA | C/CA | 0.27 |
| chr4 | 39501724 | UGDH | rs202227157 | A | C | 0.07 |
| chr4 | 39510264 | UGDH | rs1129052 | A | G | 0.39 |
| chr4 | 39515736 | UGDH | rs10019532 | A | G | 0.39 |
| chr4 | 79531308 | ANXA3 | rs4612 | C | T | 0.35 |
| chr4 | 143033778 | INPP4B | rs3822141 | T | C | 0.08 |
| chr4 | 143043340 | INPP4B | rs2270658 | A | G | 0.26 |
| chr4 | 143067053 | INPP4B | rs61733152 | C | T | 0.07 |
| chr4 | 143067054 | INPP4B | rs35390852 | G | A | 0.06 |
| chr4 | 143324094 | INPP4B | rs1982966 | G | A | 0.28 |
| chr4 | 185686032 | ACSL1 | rs2292898 | C | T | 0.08 |
| chr4 | 185687129 | ACSL1 | rs61610832 | G | A | 0.05 |
| chr4 | 185687863 | ACSL1 | rs11727009 | A | G | 0.24 |
| chr4 | 185701549 | ACSL1 | rs1803898 | G | T | 0.07 |
| chr5 | 665280 | TPPP | rs1697963 | T | C | 0.34 |
| chr5 | 678062 | TPPP | rs450628 | A | G | 0.34 |
| chr5 | 79351735 | THBS4 | rs423906 | C | T | 0.26 |
| chr5 | 79375724 | THBS4 | rs2288395 | G | C | 0.44 |
| chr5 | 141391532 | GNPDA1 | rs164080 | G | A | 0.37 |
| chr5 | 176836532 | F12 | rs1801020 | A | G | 0.28 |
| chr5 | 73985215 | HEXB | rs11556045 | A | G | 0.16 |
| chr5 | 73992881 | HEXB | rs10805890 | A | G | 0.27 |
| chr5 | 81571846 | RPS23 | rs226201 | G | A | 0.09 |
| chr5 | 81574182 | RPS23 | rs6880209 | C | T | 0.07 |
| chr5 | 82786194 | VCAN | rs12332199 | T | C | 0.18 |
| chr5 | 82789647 | VCAN | rs4470745 | A | G | 0.08 |
| chr5 | 82808072 | VCAN | rs2652098 | C | T | 0.22 |
| chr5 | 82815408 | VCAN | rs2287926 | G | A | 0.18 |
| chr5 | 82833145 | VCAN-AS1 | rs2548541 | G | A | 0.38 |
| chr5 | 82833369 | VCAN-AS1 | rs309559 | A | G | 0.36 |
| chr5 | 82833391 | VCAN-AS1 | rs16900528 | A | G | 0.06 |
| chr5 | 82834299 | VCAN-AS1 | rs188703 | G | A | 0.30 |
| chr5 | 82834630 | VCAN-AS1 | rs309557 | T | C | 0.36 |
| chr5 | 82835545 | VCAN-AS1 | rs160279 | A | G | 0.36 |
| chr5 | 82835724 | VCAN-AS1 | rs160278 | T | A | 0.36 |
| chr5 | 82836565 | VCAN-AS1 | rs75771891 | T | C | 0.06 |
| chr5 | 82837631 | VCAN-AS1 | rs160277 | G | T | 0.30 |
| chr5 | 118811533 | HSD17B4 | rs25640 | G | A | 0.48 |
| chr5 | 118861713 | HSD17B4 | rs11205 | A | G | 0.42 |
| chr5 | 118872184 | HSD17B4 | rs28943592 | C | T | 0.10 |
| chr5 | 132094205 | 8-Sep | rs17716311 | G | A | 0.10 |
| chr5 | 132099999 | 8-Sep | rs30507 | A | G | 0.23 |
| chr5 | 137892170 | HSPA9 | rs10117 | G | A | 0.21 |
| chr5 | 137902339 | HSPA9 | rs1042665 | T | C | 0.24 |
| chr5 | 137911022 | HSPA9 | rs17523658 | G | C | 0.10 |
| chr5 | 141391532 | HSPA9 | rs164080 | G | A | 0.37 |
| chr5 | 146798035 | DPYSL3 | rs17106709 | G | A | 0.21 |
| chr5 | 179126090 | CANX | rs1134924 | C | T | 0.36 |
| chr6 | 161137779 | PLG | rs14224 | T | C | 0.45 |
| chr6 | 161139480 | PLG | rs1130656 | C | T | 0.45 |
| chr6 | 161162406 | PLG | rs4252170 | T | C | 0.11 |
| chr6 | 35610590 | FKBP5 | rs146032538 | A | G | 0.06 |
| chr6 | 24495330 | ALDH5A1 | rs4646832 | G | C | 0.07 |
| chr6 | 24503590 | ALDH5A1 | rs2760118 | C | T | 0.17 |
| chr6 | 24503597 | ALDH5A1 | rs3765310 | C | T | 0.07 |
| chr7 | 127721507 | SND1 | rs322825 | T | C | 0.36 |
| chr7 | 44104788 | PGAM2 | rs61756062 | A | C | 0.06 |
| chr7 | 95214911 | PDK4 | rs55879823 | T | C | 0.05 |
| chr7 | 95216394 | PDK4 | rs4727323 | T | G | 0.18 |
| chr7 | 95216421 | PDK4 | rs11541299 | G | A | 0.11 |
| chr7 | 107599806 | LAMB1 | rs35710474 | C | T | 0.10 |
| chr7 | 107603431 | LAMB1 | rs2230156 | A | C | 0.24 |
| chr7 | 107615506 | LAMB1 | rs11770141 | C | T | 0.05 |
| chr7 | 107626770 | LAMB1 | rs25659 | G | A | 0.05 |
| chr7 | 107569517 | LAMB1 | rs1131398 | A | G | 0.45 |
| chr7 | 27702390 | HIBADH | rs11550134 | C | A | 0.47 |
| chr7 | 134143863 | AKR1B1 | rs5053 | G | C | 0.23 |
| chr7 | 44147485 | AEBP1 | rs2537188 | C | A | 0.25 |
| chr7 | 44148553 | AEBP1 | rs2595701 | A | G | 0.12 |
| chr7 | 44153780 | AEBP1 | rs13928 | A | G | 0.25 |
| chr7 | 5643145 | FSCN1 | rs1640233 | T | C | 0.16 |
| chr7 | 44924627 | PURB | rs6966024 | A | C | 0.25 |
| chr7 | 95800820 | SLC25A13 | rs2301629 | T | C | 0.41 |
| chr8 | 145729727 | GPT | rs1063739 | C | A | 0.49 |
| chr8 | 121210069 | COL14A1 | rs2305598 | T | C | 0.36 |
| chr8 | 121215991 | COL14A1 | rs2305600 | T | C | 0.35 |
| chr8 | 121228679 | COL14A1 | rs4870723 | A | C | 0.36 |
| chr8 | 121256150 | COL14A1 | rs2305603 | T | C | 0.42 |
| chr8 | 121259862 | COL14A1 | rs2305605 | G | A | 0.43 |
| chr8 | 27462481 | CLU | rs7982 | A | G | 0.20 |
| chr8 | 86360458 | CA3-AS1 | rs11340759 | TC | T/T | 0.49 |
| chr8 | 86351997 | CA3 | rs20571 | G | A | 0.43 |
| chr8 | 11710888 | CTSB | rs12338 | G | C | 0.48 |
| chr8 | 26481697 | DPYSL2 | rs2228979 | G | A | 0.10 |
| chr8 | 26481771 | DPYSL2 | rs327222 | T | C | 0.10 |
| chr8 | 26510792 | DPYSL2 | rs708621 | T | C | 0.40 |
| chr8 | 86389403 | CA2 | rs703 | T | C | 0.47 |
| chr9 | 116822510 | AMBP | rs80057939 | G | A | 0.07 |
| chr9 | 32425910 | ACO1 | rs3780473 | A | G | 0.46 |
| chr9 | 131360750 | SPTAN1 | rs2227864 | C | T | 0.49 |
| chr9 | 131379967 | SPTAN1 | rs2227862 | C | T | 0.49 |
| chr9 | 131379998 | SPTAN1 | rs3750333 | C | A | 0.10 |
| chr9 | 131866523 | CRAT | rs2228304 | C | G | 0.15 |
| chr10 | 75865065 | VCL | rs767809 | G | A | 0.26 |
| chr10 | 75871722 | VCL | rs16931179 | C | T | 0.08 |
| chr10 | 75871735 | VCL | rs2131956 | C | G | 0.37 |
| chr10 | 75406764 | SYNPO2L | rs118159842 | G | C | 0.06 |
| chr10 | 75406912 | SYNPO2L | rs34163229 | G | T | 0.19 |
| chr10 | 75407124 | SYNPO2L | rs150532364 | A | G | 0.06 |
| chr10 | 75407290 | SYNPO2L | rs3812629 | G | A | 0.19 |
| chr10 | 75407649 | SYNPO2L | rs4746139 | A | C | 0.19 |
| chr10 | 29754609 | SVIL-AS1 | rs1057952 | T | C | 0.46 |
| chr10 | 29759225 | SVIL-AS1 | rs11007607 | A | G | 0.26 |
| chr10 | 29769589 | SVIL-AS1 | rs61737920 | C | T | 0.05 |
| chr10 | 29812602 | SVIL | rs7070678 | G | T | 0.09 |
| chr10 | 29821523 | SVIL | rs1328323 | T | C | 0.08 |
| chr10 | 29822159 | SVIL | rs7076239 | T | C | 0.09 |
| chr10 | 29839787 | SVIL | rs10160013 | A | G | 0.34 |
| chr10 | 29839798 | SVIL | rs17834991 | A | G | 0.16 |
| chr10 | 29839864 | SVIL | rs1270874 | A | C | 0.15 |
| chr10 | 29840038 | SVIL | rs3740003 | A | G | 0.34 |
| chr10 | 29840164 | SVIL | rs3740002 | A | G | 0.34 |
| chr10 | 29843833 | SVIL | rs1547169 | C | T | 0.35 |
| chr10 | 16547058 | PTER | rs7904014 | A | G | 0.16 |
| chr10 | 7759595 | ITIH2 | rs7072478 | C | T | 0.22 |
| chr10 | 7780646 | ITIH2 | rs3740217 | C | G | 0.27 |
| chr10 | 92680929 | ANKRD1 | rs10881855 | A | G | 0.34 |
| chr10 | 96997589 | PDLIM1 | rs1049961 | A | G | 0.10 |
| chr10 | 96997609 | PDLIM1 | rs11872 | G | A | 0.06 |
| chr10 | 96997670 | PDLIM1 | rs1049921 | A | G | 0.08 |
| chr10 | 96997820 | PDLIM1 | rs1049814 | A | G | 0.08 |
| chr10 | 97106165 | SORBS1 | rs726176 | T | C | 0.34 |
| chr10 | 97141487 | SORBS1 | rs61739184 | G | A | 0.09 |
| chr10 | 97141523 | SORBS1 | rs2274490 | G | A | 0.37 |
| chr10 | 97174352 | SORBS1 | rs2281939 | T | C | 0.13 |
| chr10 | 97174537 | SORBS1 | rs7081076 | C | A | 0.10 |
| chr11 | 124506967 | SIAE | rs7941327 | C | T | 0.12 |
| chr11 | 124524627 | SIAE | rs1942663 | A | G | 0.12 |
| chr11 | 124539273 | SIAE | rs12282107 | T | C | 0.13 |
| chr11 | 124539295 | SIAE | rs76655561 | C | T | 0.11 |
| chr11 | 46699494 | ARHGAP1 | rs34845803 | GA | GA/GA | 0.30 |
| chr11 | 116692334 | APOA4 | rs5104 | C | T | 0.34 |
| chr11 | 837582 | CD151 | rs1130663 | G | A | 0.15 |
| chr11 | 12525925 | PARVA | rs11547363 | C | T | 0.17 |
| chr11 | 77553638 | AAMDC | rs585721 | T | C | 0.25 |
| chr11 | 77583266 | AAMDC | rs2186564 | G | A | 0.17 |
| chr11 | 77583376 | AAMDC | rs588217 | G | A | 0.08 |
| chr11 | 111781047 | CRYAB | rs11603779 | A | C | 0.16 |
| chr11 | 122928622 | HSPA8 | rs4802 | A | G | 0.43 |
| chr11 | 126174038 | DCPS | rs3740915 | C | T | 0.13 |
| chr11 | 126174164 | DCPS | rs695029 | C | T | 0.31 |
| chr11 | 126201403 | DCPS | rs616360 | T | C | 0.15 |
| chr12 | 49582692 | TUBA1A | rs1039225 | T | G | 0.26 |
| chr12 | 48501161 | PFKM | rs11609399 | A | T | 0.44 |
| chr12 | 48526712 | PFKM | rs2228500 | G | A | 0.29 |
| chr12 | 48527186 | PFKM | rs1049392 | C | T | 0.14 |
| chr12 | 91502250 | LUM | rs17853500 | A | G | 0.31 |
| chr12 | 7637795 | CD163 | rs79148200 | A | G | 0.06 |
| chr12 | 7649484 | CD163 | rs4883263 | T | C | 0.31 |
| chr12 | 7649653 | CD163 | rs4883264 | T | C | 0.37 |
| chr12 | 7655137 | CD163 | rs3210140 | A | G | 0.37 |
| chr12 | 81627238 | ACSS3 | rs1921038 | C | T | 0.24 |
| chr12 | 6976688 | TPI1 | rs1800200 | A | G | 0.25 |
| chr12 | 49332366 | ARF3 | rs4760666 | C | T | 0.50 |
| chr12 | 49332451 | ARF3 | rs148694584 | T | TGA/TGA | 0.48 |
| chr12 | 57926791 | DCTN2 | rs2292656 | T | G | 0.19 |
| chr12 | 95906603 | METAP2 | rs3794261 | C | A | 0.17 |
| chr12 | 106633569 | CKAP4 | rs3088113 | C | T | 0.42 |
| chr14 | 94772504 | SERPINA6 | rs1042394 | G | A | 0.05 |
| chr14 | 94776219 | SERPINA6 | rs2228542 | C | T | 0.05 |
| chr14 | 94780608 | SERPINA6 | rs3748320 | G | A | 0.08 |
| chr14 | 95033356 | SERPINA4 | rs5510 | C | T | 0.28 |
| chr14 | 95029852 | SERPINA4 | rs5508 | G | T | 0.12 |
| chr14 | 95080803 | SERPINA3 | rs4934 | G | A | 0.39 |
| chr14 | 24615435 | PSME2 | rs4575 | T | C | 0.37 |
| chr14 | 52478315 | NID2 | rs1051069 | A | G | 0.23 |
| chr14 | 52481917 | NID2 | rs946615 | C | T | 0.32 |
| chr14 | 52505517 | NID2 | rs2273432 | G | A | 0.24 |
| chr14 | 52507429 | NID2 | rs3742536 | A | G | 0.17 |
| chr14 | 52509501 | NID2 | rs3818186 | T | C | 0.48 |
| chr14 | 52520368 | NID2 | rs2101919 | C | T | 0.48 |
| chr14 | 23859610 | MYH6 | rs28730771 | C | T | 0.09 |
| chr14 | 23861811 | MYH6 | rs365990 | A | G | 0.17 |
| chr14 | 23874507 | MYH6 | rs2277473 | G | T | 0.06 |
| chr14 | 23874523 | MYH6 | rs2277474 | C | T | 0.06 |
| chr14 | 55604710 | LGALS3 | rs10606761 | TCTG | TT | 0.08 |
| chr14 | 55605036 | LGALS3 | rs4652 | A | C | 0.40 |
| chr14 | 65008251 | HSPA2 | rs1063391 | C | T | 0.13 |
| chr14 | 102568367 | HSP90AA1 | rs8005905 | T | A | 0.19 |
| chr14 | 102550803 | HSP90AA1 | rs4947 | G | A | 0.21 |
| chr14 | 69352230 | ACTN1 | rs15993 | G | A | 0.16 |
| chr14 | 20925154 | APEX1 | rs1130409 | T | G | 0.40 |
| chr14 | 100808845 | WARS | rs9453 | G | A | 0.21 |
| chr14 | 105196365 | ADSSL1 | rs33958252 | T | C | 0.37 |
| chr14 | 105211221 | ADSSL1 | rs12432802 | C | T | 0.47 |
| chr14 | 105213343 | ADSSL1 | rs6644 | A | G | 0.35 |
| chr15 | 99645813 | SYNM | rs1044356 | G | A | 0.37 |
| chr15 | 99645862 | SYNM | rs11014 | A | C | 0.37 |
| chr15 | 99653800 | SYNM | rs2305445 | T | C | 0.29 |
| chr15 | 99669628 | SYNM | rs3743242 | A | T | 0.30 |
| chr15 | 99670161 | SYNM | rs3134586 | C | T | 0.30 |
| chr15 | 99670278 | SYNM | rs3134587 | C | T | 0.30 |
| chr15 | 99670518 | SYNM | rs1670227 | T | C | 0.07 |
| chr15 | 99670989 | SYNM | rs3743248 | T | C | 0.28 |
| chr15 | 99671760 | SYNM | rs260087 | T | C | 0.27 |
| chr15 | 99672722 | SYNM | rs2292288 | A | G | 0.34 |
| chr15 | 89450546 | MFGE8 | rs1878327 | C | T | 0.46 |
| chr15 | 89450587 | MFGE8 | rs1878326 | G | T | 0.45 |
| chr15 | 89456544 | MFGE8 | rs4945 | G | T | 0.12 |
| chr15 | 90628537 | IDH2 | rs11540478 | G | A | 0.19 |
| chr15 | 45654327 | GATM | rs1145086 | A | G | 0.06 |
| chr15 | 45661678 | GATM | rs1288775 | T | A | 0.15 |
| chr15 | 30008889 | TJP1 | rs2229518 | G | A | 0.16 |
| chr15 | 30018627 | TJP1 | rs2229515 | T | C | 0.12 |
| chr15 | 62990971 | TLN2 | rs12905981 | C | T | 0.35 |
| chr15 | 63009804 | TLN2 | rs1320191 | C | T | 0.06 |
| chr15 | 63047778 | TLN2 | rs8033767 | C | T | 0.33 |
| chr15 | 63047793 | TLN2 | rs4775538 | C | T | 0.28 |
| chr15 | 63111739 | TLN2 | rs3816988 | T | C | 0.09 |
| chr15 | 63127983 | TLN2 | rs8038919 | G | A | 0.10 |
| chr15 | 63131117 | TLN2 | rs937418 | T | A | 0.34 |
| chr15 | 63433785 | LACTB | rs4775629 | G | A | 0.17 |
| chr16 | 1877494 | FAHD1 | rs3743854 | T | C | 0.16 |
| chr16 | 1877558 | FAHD1 | rs3743853 | G | A | 0.16 |
| chr16 | 1877913 | FAHD1 | rs13166 | C | G | 0.10 |
| chr16 | 8841960 | ABAT | rs1641010 | T | C | 0.43 |
| chr16 | 4242028 | SRL | rs10852642 | A | T | 0.42 |
| chr16 | 4242093 | SRL | rs8051399 | C | T | 0.41 |
| chr16 | 4245693 | SRL | rs10852643 | A | G | 0.30 |
| chr17 | 42335144 | SLC4A1 | rs13306781 | C | T | 0.07 |
| chr17 | 42338945 | SLC4A1 | rs5036 | T | C | 0.07 |
| chr17 | 42338998 | SLC4A1 | rs5035 | T | G | 0.09 |
| chr17 | 1674429 | SERPINF1 | rs8074840 | T | C | 0.33 |
| chr17 | 1680002 | SERPINF1 | rs6828 | T | C | 0.21 |
| chr17 | 1673276 | SERPINF1 | rs1136287 | C | T | 0.43 |
| chr17 | 74684858 | MXRA7 | rs7219390 | T | C | 0.33 |
| chr17 | 4856376 | ENO3 | rs238238 | A | G | 0.36 |
| chr17 | 4856580 | ENO3 | rs238239 | T | C | 0.16 |
| chr17 | 73127110 | NT5C | rs3736076 | A | T | 0.06 |
| chr17 | 73127683 | NT5C | rs4788867 | T | C | 0.30 |
| chr18 | 72163592 | CNDP2 | rs6566811 | G | A | 0.29 |
| chr18 | 72168608 | CNDP2 | rs2303463 | G | A | 0.27 |
| chr18 | 72176083 | CNDP2 | rs2278161 | T | C | 0.28 |
| chr18 | 72178161 | CNDP2 | rs2278159 | T | C | 0.28 |
| chr18 | 72187334 | CNDP2 | rs111384710 | G | A | 0.10 |
| chr18 | 12308075 | TUBB6 | rs117110301 | G | C | 0.12 |
| chr18 | 12308243 | TUBB6 | rs8087840 | T | A | 0.41 |
| chr18 | 12308245 | TUBB6 | rs8086821 | C | T | 0.41 |
| chr18 | 12308273 | TUBB6 | rs11548177 | G | T | 0.15 |
| chr19 | 10736237 | SLC44A2 | rs3745242 | C | G | 0.44 |
| chr19 | 10736267 | SLC44A2 | rs3745241 | G | A | 0.31 |
| chr19 | 10738639 | SLC44A2 | rs3087969 | C | T | 0.36 |
| chr19 | 10742170 | SLC44A2 | rs2288904 | A | G | 0.36 |
| chr19 | 55614923 | PPP1R12C | rs2532060 | T | C | 0.25 |
| chr19 | 55624113 | PPP1R12C | rs34521018 | G | A | 0.13 |
| chr19 | 55628609 | PPP1R12C | rs66707428 | A | G | 0.25 |
| chr19 | 15582863 | PGLYRP2 | rs34440547 | C | T | 0.29 |
| chr19 | 15586672 | PGLYRP2 | rs892145 | A | T | 0.34 |
| chr19 | 15587185 | PGLYRP2 | rs733731 | C | T | 0.34 |
| chr19 | 15587345 | PGLYRP2 | rs3813135 | T | C | 0.34 |
| chr19 | 36246418 | HSPB6 | rs11549030 | C | G | 0.09 |
| chr19 | 830854 | AZU1 | rs595844 | T | C | 0.44 |
| chr19 | 45409167 | APOE | rs440446 | C | G | 0.40 |
| chr19 | 45411941 | APOE | rs429358 | T | C | 0.10 |
| chr19 | 45412079 | APOE | rs7412 | C | T | 0.08 |
| chr19 | 3983184 | EEF2 | rs2230560 | A | G | 0.07 |
| chr19 | 4538599 | LRG1 | rs966384 | G | A | 0.46 |
| chr19 | 6677989 | C3 | rs17030 | G | A | 0.44 |
| chr19 | 6681991 | C3 | rs7951 | G | A | 0.09 |
| chr19 | 6697406 | C3 | rs423490 | A | G | 0.07 |
| chr19 | 6702157 | C3 | rs428453 | C | G | 0.13 |
| chr19 | 6709704 | C3 | rs2230205 | C | T | 0.47 |
| chr19 | 6713291 | C3 | rs2230201 | C | T | 0.48 |
| chr19 | 33878197 | PEPD | rs77690463 | G | A | 0.06 |
| chr19 | 33878198 | PEPD | rs1061338 | A | G | 0.23 |
| chr19 | 33878837 | PEPD | rs17570 | G | A | 0.23 |
| chr19 | 33878977 | PEPD | rs2230062 | C | T | 0.10 |
| chr19 | 33882222 | PEPD | rs17569 | G | A | 0.24 |
| chr19 | 33953912 | PEPD | rs3745969 | A | G | 0.08 |
| chr19 | 39196745 | ACTN4 | rs3745859 | C | T | 0.43 |
| chr19 | 39219780 | ECH1 | rs1136956 | T | C | 0.06 |
| chr19 | 39306529 | ECH1 | rs58750431 | C | T | 0.11 |
| chr19 | 39307103 | ECH1 | rs2229259 | C | T | 0.08 |
| chr19 | 39308190 | ECH1 | rs15570 | C | T | 0.12 |
| chr19 | 39322087 | ECH1 | rs9419 | T | G | 0.26 |
| chr19 | 50140092 | RRAS | rs1865077 | G | A | 0.17 |
| chr20 | 5166418 | CDS2 | rs3818196 | G | A | 0.08 |
| chr20 | 5169799 | CDS2 | rs34096549 | C | T | 0.09 |
| chr20 | 36766400 | TGM2 | rs2281196 | G | A | 0.06 |
| chr20 | 36770588 | TGM2 | rs2076390 | C | T | 0.34 |
| chr20 | 36974945 | LBP | rs2232580 | C | T | 0.08 |
| chr20 | 36977970 | LBP | rs1739654 | G | A | 0.16 |
| chr20 | 36979265 | LBP | rs2232582 | T | C | 0.08 |
| chr20 | 36989381 | LBP | rs2232596 | G | A | 0.14 |
| chr20 | 37001761 | LBP | rs2232618 | T | C | 0.07 |
| chr20 | 42743454 | JPH2 | rs6093935 | A | G | 0.09 |
| chr20 | 42744587 | JPH2 | rs74352869 | G | C | 0.07 |
| chr20 | 42747247 | JPH2 | rs3810510 | C | T | 0.38 |
| chr20 | 42815190 | JPH2 | rs1883790 | G | A | 0.25 |
| chr20 | 43530234 | YWHAB | rs4931 | A | C | 0.12 |
| chr20 | 62126185 | EEF1A2 | rs310617 | A | G | 0.36 |
| chr20 | 62126299 | EEF1A2 | rs2274860 | C | T | 0.43 |
| chr20 | 62127326 | EEF1A2 | rs3818681 | G | A | 0.11 |
| chr21 | 46876083 | COL18A1 | rs2236451 | A | G | 0.35 |
| chr21 | 46876173 | COL18A1 | rs2236452 | C | T | 0.18 |
| chr21 | 46876306 | COL18A1 | rs11702494 | G | A | 0.14 |
| chr21 | 46876580 | COL18A1 | rs8133886 | C | T | 0.46 |
| chr21 | 46876717 | COL18A1 | rs77180157 | G | C | 0.08 |
| chr21 | 46896294 | COL18A1 | rs2230686 | G | A | 0.07 |
| chr21 | 46896303 | COL18A1 | rs2230687 | C | G | 0.28 |
| chr21 | 46897801 | COL18A1 | rs2230689 | G | A | 0.07 |
| chr21 | 46899842 | COL18A1 | rs1131100 | C | T | 0.09 |
| chr21 | 46899857 | COL18A1 | rs1131101 | C | T | 0.09 |
| chr21 | 46900410 | COL18A1 | rs2236467 | C | T | 0.09 |
| chr21 | 46911188 | COL18A1 | rs79980197 | C | G | 0.08 |
| chr21 | 46913477 | COL18A1 | rs2230693 | G | C | 0.08 |
| chr21 | 46929467 | COL18A1 | rs1050351 | G | A | 0.46 |
| chr21 | 45553596 | C21or f33 | rs968714 | T | C | 0.28 |
| chr21 | 45564766 | C21or f33 | rs2838497 | C | G | 0.11 |
| chr21 | 45565473 | C21orf33 | rs7676 | C | T | 0.17 |
| chr22 | 31494813 | SMTN | rs80055673 | G | C | 0.05 |
| chr22 | 19950235 | COMT | rs4633 | C | T | 0.27 |
| chr22 | 19950263 | COMT | rs6267 | G | T | 0.06 |
| chr22 | 19951207 | COMT | rs4818 | C | G | 0.33 |
| chr22 | 19951271 | COMT | rs4680 | G | A | 0.27 |
| chr22 | 19951804 | COMT | rs769224 | G | A | 0.06 |
| chr22 | 18209613 | BCL2L13 | rs4488761 | A | G | 0.09 |
| chr22 | 24581207 | SUSD2 | rs3752497 | C | T | 0.11 |
| chr22 | 24582041 | SUSD2 | rs8141797 | A | G | 0.11 |
| chr22 | 36007045 | MB | rs7292 | G | A | 0.25 |
| chr22 | 36007075 | MB | rs7293 | C | T | 0.24 |
| chr22 | 44351351 | SAMM50 | rs2294920 | T | C | 0.09 |
| chr22 | 44368122 | SAMM50 | rs3761472 | A | G | 0.39 |
| chr22 | 44368204 | SAMM50 | rs3177036 | A | G | 0.20 |
| chr22 | 44372632 | SAMM50 | rs14315 | C | T | 0.48 |
| chr22 | 44379838 | SAMM50 | rs8418 | A | G | 0.20 |
| chr22 | 44386281 | SAMM50 | rs7587 | C | T | 0.33 |
| **# common variant in gene was defined maf >0.05** | | | | | | |

| **S3 Table Association of GRS group and heart failure** | | | | | | | |
| --- | --- | --- | --- | --- | --- | --- | --- |
| **Group** | **Number** | **Incident Cases** | **model** | **p valul** | **HR** | **95.0% CI** | |
| Tertiles | 1000 | 260 | ^†^T-1 | 3.75×10^-10^ | 1.89 | 1.55 | 2.31 |
|  |  |  | ^※^T-2 | 6.12×10^-11^ | 1.95 | 1.60 | 2.39 |
|  |  |  | ^＊^T-3 | 5.07×10^-11^ | 1.96 | 1.60 | 2.39 |
| Quartiles | 1000 | 260 | ^†^Q-1 | 4.91×10^-9^ | 1.69 | 1.42 | 2.02 |
|  |  |  | ^※^Q-2 | 2.00×10^-9^ | 1.71 | 1.44 | 2.04 |
|  |  |  | ^＊^Q-3 | 1.48×10^-9^ | 1.72 | 1.45 | 2.06 |
| ^†^Association tested with Cox proportional hazards original risk model. | | | | | | | |
| ^※^Association tested with Cox proportional hazards model adjusted gender and age. | | | | | | | |
| ^＊^Association tested with Cox proportional hazards model adjusted gender, age and traditional risk factor: hypertension, hyperlipemia, diabetes mellitus, current smoking. | | | | | | | |

| **S4 Table Relationship of Genetic risk score and prognosis of Heart failure** | | | | | | | | | | |
| --- | --- | --- | --- | --- | --- | --- | --- | --- | --- | --- |
|  | **Group** | **GRS** | **Number** | **Incident Cases** | **unadjust** | | | **adjust** | | |
|  |  |  |  |  | **P Value** | **HR** | **95%CI** | **P Value** | **HR** | **95%CI** |
| Tertiles | Lower | -3~0 | 338 | 52 | - | 1.00 | - | - | 1.00 | - |
|  | Middler | 1~3 | 462 | 167 | ＊8.00×10^-6^ | 2.04 | 1.49-2.79 | ^＊^4.00×10^-6^ | 2.15 | 1.55-2.97 |
|  | Upper | 4~7 | 185 | 41 | ^※^1.34×10^-9^ | 3.56 | 2.36-5.37 | ^※^2.47×10^-10^ | 3.68 | 2.40-5.65 |
| Quartiles | Q1 | -3~-1 | 134 | 15 | - | 1.00 | - | - | 1.00 | - |
|  | Q2 | 0~1 | 422 | 99 | ^†^1.706×10^-3^ | 2.39 | 1.39-4.13 | ^†^2.09×10-^3^ | 2.36 | 1.37-4.07 |
|  | Q3 | 2~4 | 421 | 133 | ^‡^5.00×10^-6^ | 3.50 | 2.05-5.99 | ^‡^3.00×10^-6^ | 3.61 | 2.11-6.17 |
|  | Q4 | 5~7 | 24 | 13 | **^＃^**3.44×10^-7^ | 6.96 | 3.30-14.68 | **^＃^**5.27×10^-7^ | 6.76 | 3.21-14.28 |
| GRS=genetic risk score. Association tested with Cox proportional hazards model adjusted for gender,age, hypertension,hyperlipemia, diabetes mellitus, current smoking, β-blocker treatment. ＊middler VS lower, ※upper VS lower,†Q2 VS Q1,‡Q3 VS Q1,＃Q4 VS Q1 | | | | | | | | | | |
|  |  |  |  |  |  |  |  |  |  |  |

| **S5 Table AUC for HF with different models** | | | | |
| --- | --- | --- | --- | --- |
| Models | Variable | AUC | SE | 95% CI |
| Individuals traditonal risk factor | Gender | 0.529 | 0.0163 | 0.498 to 0.560 |
|  | Age | 0.626 | 0.02 | 0.595to 0.656 |
|  | DM2 | 0.527 | 0.0144 | 0.496 to 0.558 |
|  | Hyperlipemia | 0.502 | 0.0077 | 0.470 to 0.533 |
|  | Hypertension | 0.509 | 0.0176 | 0.477 to 0.540 |
|  | Current somking | 0.513 | 0.0178 | 0.482 to 0.545 |
|  | GRS | 0.620 | 0.0194 | 0.589 to 0.650 |
|  | NT-proBNP | 0.721 | 0.0219 | 0.685 to 0.755 |
| Combined risk factor | NT-proBNP+GRS | 0.754 | 0.0209 | 0.720 to 0.787 |
|  | TRA | 0.648 | 0.0243 | 0.610 to 0.684 |
|  | TRA+GRS | 0.690 | 0.0233 | 0.653 to 0.725 |
|  | NT-proBNP+TRA | 0.748 | 0.0215 | 0.713 to 0.781 |
|  | NT-proBNP+TRA+GRS | 0.773 | 0.0206 | 0.740 to 0.804 |
| Tra=traditonal risk factor,GRS=genetic risk score. | | | | |

| **S6 Table C-index data compared different model** | | | | | |
| --- | --- | --- | --- | --- | --- |
| **Components** | **Difference between areas** | **Standard Error** | **95% CI** | **z statistic** | **Significance level** |
| GRS VS Age | 0.0587 | 0.0287 | -0.0503 to 0.0621 | 0.205 | P = 0.8377 |
| GRS VS DM2 | 0.0928 | 0.0239 | 0.0460 to 0.140 | 3.886 | P = 0.0001 |
| GRS VS Gender | 0.0908 | 0.0253 | 0.0413 to 0.140 | 3.592 | P = 0.0003 |
| GRS VS Hyperlipemia | 0.1180 | 0.0211 | 0.0765 to 0.159 | 5.580 | P < 0.0001 |
| GRS VS Hypertension | 0.1110 | 0.0262 | 0.0600 to 0.163 | 4.251 | P < 0.0001 |
| GRS VS Current smoking | 0.1160 | 0.0263 | 0.0648 to 0.168 | 4.428 | P < 0.0001 |
| GRS **VS** NT-proBNP | 0.0918 | 0.0329 | 0.0272 to 0.156 | 3.791 | P = 0.0001 |
| GRS **VS** TRA | 0.0183 | 0.0341 | -0.0486 to 0.852 | 0.536 | P = 0.5921 |
| GRS VS TRA+GRS | 0.0607 | 0.0202 | 0.0212 to 0.100 | 3.008 | P = 0.0026 |
| GRS **VS** NT-proBNP+TRA | 0.1190 | 0.0325 | 0.0553 to 0.183 | 3.661 | P = 0.0003 |
| GRS **VS** NT-proBNP+TRA+GRS | 0.1440 | 0.0241 | 0.0967to 0.191 | 5.970 | P < 0.0001 |
| NT-proBNP **VS** TRA | 0.0734 | 0.0305 | 0.0136 to 0.133 | 2.406 | P = 0.0161 |
| NT-proBNP VS TRA+GRS | 0.0310 | 0.0304 | -0.0286 to 0.0385 | 1.019 | P = 0.3083 |
| NT-proBNP **VS** NT-proBNP+TRA | 0.0274 | 0.0127 | 0.00241 to 0.0524 | 2.149 | P = 0.0316 |
| NT-proBNP VS NT-proBNP+TRA+GRS | 0.0522 | 0.0165 | 0.0198 to 0.0847 | 3.158 | P = 0.0016 |
| TRA VS TRA+GRS | 0.0424 | 0.0178 | 0.00759 to 0.0773 | 2.387 | P = 0.0170 |
| TRA **VS** NT-proBNP+TRA | 0.1010 | 0.0212 | 0.0593 to 0.142 | 4.762 | P < 0.0001 |
| TRA **VS** NT-proBNP+TRA+GRS | 0.1260 | 0.0228 | 0.0811 to 0.170 | 5.232 | P < 0.0001 |
| TRA+GRS VS NT-proBNP+TRA | 0.0584 | 0.024 | 0.0114 to 0.105 | 2.437 | P = 0.0148 |
| TRA+GRS VS NT-proBNP+TRA+GRS | 0.0832 | 0.0176 | 0.0488 to 0.118 | 4.736 | P < 0.0001 |
| NT-proBNP+TRA**VS** NT-proBNP+TRA+GRS | 0.0248 | 0.0115 | 0.00240 - 0.0473 | 2.619 | P = 0.0301 |
| Tra=traditional risk factor, GRS=genetic risk score, NT-proBNP=N-terminal B-type natriuretic peptide. | | | | | |

| **S7 Table. Haploblock Structure within Gene** | | | |
| --- | --- | --- | --- |
| **Haploblock Structure** | **gene** | **SNP** | **Chromosome** |
| Haploblock 1 | SYNM | rs3134586 | Chr15:99129956 |
|  | SYNM | rs3134587 | Chr15:99130073 |
|  | SYNM | rs3743242 | Chr15:99129423 |
| SNPs in each haploblock are in strong linkage disequilibrium (r2≥0.9). SNPs = single nucleotide polymorphisms. | | | |
|  |  |  |  |

| **S8 Table8 NT-proBNP in GRS models** | | | | | |
| --- | --- | --- | --- | --- | --- |
|  | **Group** | **GRS** | **N** | **ln（NT-proBNP）** | **P value** |
| Tertiles | Lower | -4~-1 | 338 | 3.43±0.62 | p=0.895 |
|  | Middler | 0~1 | 452 | 3.45±0.69 |  |
|  | Upper | 2~5 | 185 | 3.45±0.66 |  |
| Quartiles | Q1 | -4~-2 | 144 | 3.48±0.56 | p=0.704 |
|  | Q2 | -1~0 | 442 | 3.41±0.70 |  |
|  | Q3 | 1~2 | 340 | 3.47±0.59 |  |
|  | Q4 | 3~5 | 59 | 3.45±0.67 |  |

**S1 Figure: Flowchart of the study**


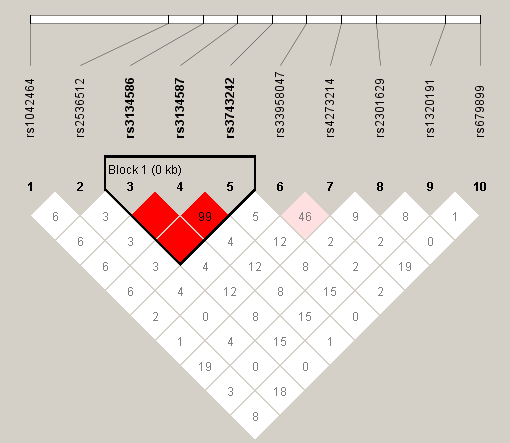
**S2 Figure: LD Structure and Haplotype Blocks of the 10 SNPs**

Supplementary References

1. Hunt SA, Abraham WT, Chin MH, Feldman AM, Francis GS, Ganiats TG, Jessup M, Konstam MA, Mancini DM, Michl K, Oates JA, Rahko PS, Silver MA, Stevenson LW, Yancy CW. 2009 Focused Update Incorporated Into the ACC/AHA 2005 Guidelines for the Diagnosis and Management of Heart Failure in Adults. *Journal of the American College of Cardiology*. 2009;53:e1-e90.

2. Report of the 1995 World Health Organization[PMIDJ8598070].

3. Chen CY, Caporizzo MA, Bedi K, Vite A, Bogush AI, Robison P, Heffler JG, Salomon AK, Kelly NA, Babu A, Morley MP, Margulies KB, Prosser BL. Suppression of detyrosinated microtubules improves cardiomyocyte function in human heart failure. *Nat Med*. 2018;24:1225–1233.
